# Supplementary material for: Topical Diacerein Decreases Skin and Splenic CD11c+ Dendritic Cells in Psoriasis
Source: Int J Mol Sci. 2023 Feb 21;24(5):4324. doi: 10.3390/ijms24054324 (PMC10001455; doi:10.3390/ijms24054324)
Supplement: Supplementary file 1 [file ijms-24-04324-s001.zip › ijms-2122866-supplementary.pdf]

SUPPLEMENTARY TABLES

Supplementary Table S1. Semiquantitative scores of erythema per day per treatment group. Means with standard deviation (SD) and group sizes (n) are shown.

	IMQ + none			IMQ + Placebo			IMQ + 2.5%			IMQ + 5%			IMQ + 10%		
**Days**	**Mean**	**SD**	**n**	**Mean**	**SD**	**n**	**Mean**	**SD**	**n**	**Mean**	**SD**	**n**	**Mean**	**SD**	**n**
**1**	0.00	0.00	3	0.00	0.00	9	0.00	0.00	9	0.00	0.00	9	0.00	0.00	9
**2**	0.00	0.00	3	0.00	0.00	9	0.00	0.00	9	0.03	0.08	9	0.03	0.08	9
**3**	0.08	0.14	3	0.11	0.22	9	0.14	0.22	9	0.17	0.25	9	0.28	0.26	9
**4**	0.92	0.14	3	0.28	0.26	9	0.39	0.47	9	0.53	0.32	9	0.42	0.35	9
**5**	1.08	0.14	3	0.67	0.30	6	0.63	0.54	6	0.79	0.33	6	0.71	0.19	6
**6**	1.25	0.25	3	0.83	0.38	6	0.75	0.45	6	0.71	0.40	6	0.88	0.47	6
**7**	0.67	0.29	3	0.79	0.58	6	0.46	0.10	6	0.54	0.25	6	0.63	0.31	6

	Vehicle + none			Vehicle + Placebo			Vehicle + 2.5%			Vehicle + 5%			Vehicle + 10%		
**Days**	**Mean**	**SD**	**n**	**Mean**	**SD**	**n**	**Mean**	**SD**	**n**	**Mean**	**SD**	**n**	**Mean**	**SD**	**n**
**1**	0.00	0.00	3	0.00	0.00	9	0.00	0.00	9	0.00	0.00	9	0.00	0.00	9
**2**	0.00	0.00	3	0.00	0.00	9	0.00	0.00	9	0.00	0.00	9	0.06	0.17	9
**3**	0.00	0.00	3	0.19	0.24	9	0.11	0.18	9	0.06	0.17	9	0.03	0.08	9
**4**	0.00	0.00	3	0.14	0.22	9	0.14	0.22	9	0.11	0.18	9	0.08	0.18	9
**5**	0.00	0.00	3	0.17	0.20	6	0.17	0.26	6	0.00	0.00	6	0.17	0.20	6
**6**	0.00	0.00	3	0.00	0.00	6	0.00	0.00	6	0.00	0.00	6	0.00	0.00	6
**7**	0.00	0.00	3	0.00	0.00	6	0.17	0.26	6	0.00	0.00	6	0.00	0.00	6

Supplementary Table S2. Semiquantitative scores of scaling per day per treatment group. Means with standard deviation (SD) and group sizes (n) are shown.

	IMQ + none			IMQ + Placebo			IMQ + 2.5%			IMQ + 5%			IMQ + 10%		
**Days**	**Mean**	**SD**	**n**	**Mean**	**SD**	**n**	**Mean**	**SD**	**n**	**Mean**	**SD**	**n**	**Mean**	**SD**	**n**
**1**	0.00	0.00	3	0.00	0.00	9	0.00	0.00	9	0.00	0.00	9	0.00	0.00	9
**2**	0.17	0.29	3	0.06	0.17	9	0.00	0.00	9	0.00	0.00	9	0.00	0.00	9
**3**	0.58	0.14	3	0.11	0.22	9	0.14	0.22	9	0.11	0.22	9	0.33	0.35	9
**4**	1.08	0.14	3	0.75	0.53	9	0.53	0.29	9	0.64	0.40	9	0.56	0.51	9
**5**	1.67	0.29	3	0.88	0.38	6	0.79	0.33	6	1.25	0.59	6	0.54	0.60	6
**6**	1.92	1.01	3	1.08	0.56	6	0.92	0.26	6	1.13	0.38	6	0.83	0.41	6
**7**	1.33	0.58	3	1.00	0.69	6	0.75	0.22	6	0.92	0.58	6	1.00	0.47	6
	**Vehicle + none**			**Vehicle + Placebo**			**Vehicle + 2.5%**			**Vehicle + 5%**			**Vehicle + 10%**		
**Days**	**Mean**	**SD**	**n**	**Mean**	**SD**	**n**	**Mean**	**SD**	**n**	**Mean**	**SD**	**n**	**Mean**	**SD**	**n**
**1**	0.00	0.00	3	0.00	0.00	9	0.00	0.00	9	0.00	0.00	9	0.00	0.00	9
**2**	0.08	0.14	3	0.08	0.18	9	0.14	0.22	9	0.06	0.11	9	0.03	0.08	9
**3**	0.42	0.14	3	0.33	0.22	9	0.22	0.23	9	0.17	0.22	9	0.19	0.24	9
**4**	0.50	0.00	3	0.72	0.51	9	0.47	0.23	9	0.28	0.34	9	0.31	0.27	9
**5**	0.50	0.00	3	0.83	0.30	6	0.54	0.25	6	0.67	0.20	6	0.79	0.25	6
**6**	0.50	0.00	3	0.50	0.16	6	0.42	0.20	6	0.67	0.20	6	0.50	0.32	6
**7**	0.33	0.29	3	0.33	0.44	6	0.38	0.44	6	0.25	0.27	6	0.29	0.40	6

Supplementary Table S3. Semiquantitative scores of skin thickening per day per treatment group. Means with standard deviation (SD) and group sizes (n) are shown.

	IMQ + none			IMQ + Placebo			IMQ + 2.5%			IMQ + 5%			IMQ + 10%		
**Days**	**Mean**	**SD**	**n**	**Mean**	**SD**	**n**	**Mean**	**SD**	**n**	**Mean**	**SD**	**n**	**Mean**	**SD**	**n**
**1**	0.00	0.00	3	0.00	0.00	9	0.00	0.00	9	0.00	0.00	9	0.00	0.00	9
**2**	0.42	0.14	3	0.42	0.31	9	0.28	0.23	9	0.31	0.35	9	0.44	0.17	9
**3**	1.00	0.00	3	0.92	0.31	9	0.81	0.24	9	0.86	0.33	9	0.94	0.27	9
**4**	1.58	0.52	3	1.33	0.35	9	1.03	0.26	9	1.31	0.39	9	1.19	0.24	9
**5**	1.58	0.80	3	1.42	0.34	6	1.29	0.29	6	1.38	0.59	6	1.67	0.30	6
**6**	1.50	0.87	3	1.54	0.25	6	1.17	0.30	6	1.08	0.20	6	1.58	0.38	6
**7**	1.00	0.50	3	1.17	0.26	6	0.79	0.25	6	1.08	0.20	6	1.04	0.25	6

	Vehicle + none			Vehicle + Placebo			Vehicle + 2.5%			Vehicle + 5%			Vehicle + 10%		
**Days**	**Mean**	**SD**	**n**	**Mean**	**SD**	**n**	**Mean**	**SD**	**n**	**Mean**	**SD**	**n**	**Mean**	**SD**	**n**
**1**	0.00	0.00	3	0.00	0.00	9	0.00	0.00	9	0.00	0.00	9	0.00	0.00	9
**2**	0.42	0.14	3	0.25	0.25	9	0.19	0.24	9	0.17	0.22	9	0.22	0.26	9
**3**	0.58	0.14	3	0.58	0.18	9	0.42	0.31	9	0.33	0.22	9	0.50	0.22	9
**4**	0.50	0.00	3	0.72	0.23	9	0.67	0.31	9	0.72	0.34	9	0.64	0.22	9
**5**	0.75	0.25	3	0.96	0.10	6	0.63	0.31	6	0.79	0.46	6	0.92	0.34	6
**6**	0.75	0.25	3	0.67	0.20	6	0.79	0.33	6	0.67	0.20	6	1.00	0.27	6
**7**	0.42	0.14	3	0.54	0.40	6	0.71	0.25	6	0.46	0.19	6	0.58	0.34	6

Supplementary Table S4. Cumulative semiquantitative scores of clinical severity per day per treatment group. Means with standard deviation (SD) and group sizes (n) are shown.

	IMQ + none			IMQ + Placebo			IMQ + 2.5%			IMQ + 5%			IMQ + 10%		
**Days**	**Mean**	**SD**	**n**	**Mean**	**SD**	**n**	**Mean**	**SD**	**n**	**Mean**	**SD**	**n**	**Mean**	**SD**	**n**
**1**	0.00	0.00	3	0.00	0.00	9	0.00	0.00	9	0.00	0.00	9	0.00	0.00	9
**2**	0.58	0.38	3	0.47	0.36	9	0.28	0.23	9	0.33	0.38	9	0.47	0.20	9
**3**	1.67	0.29	3	1.14	0.63	9	1.08	0.41	9	1.14	0.55	9	1.56	0.69	9
**4**	3.58	0.52	3	2.36	0.57	9	1.94	0.75	9	2.47	0.84	9	2.17	0.52	9
**5**	4.33	1.01	3	2.96	0.51	6	2.71	0.91	6	3.42	1.29	6	2.92	0.65	6
**6**	4.67	1.91	3	3.46	0.98	6	2.83	0.66	6	2.92	0.68	6	3.29	0.97	6
**7**	3.00	0.87	3	2.96	1.48	6	2.00	0.32	6	2.54	0.98	6	2.67	0.72	6

	Vehicle + none			Vehicle + Placebo			Vehicle + 2.5%			Vehicle + 5%			Vehicle + 10%		
**Days**	**Mean**	**SD**	**n**	**Mean**	**SD**	**n**	**Mean**	**SD**	**n**	**Mean**	**SD**	**n**	**Mean**	**SD**	**n**
**1**	0.00	0.00	3	0.00	0.00	9	0.00	0.00	9	0.00	0.00	9	0.00	0.00	9
**2**	0.50	0.25	3	0.33	0.28	9	0.33	0.33	9	0.22	0.32	9	0.31	0.43	9
**3**	1.00	0.25	3	1.11	0.47	9	0.75	0.45	9	0.56	0.33	9	0.72	0.38	9
**4**	1.00	0.00	3	1.58	0.60	9	1.28	0.59	9	1.11	0.28	9	1.03	0.38	9
**5**	1.25	0.25	3	1.96	0.43	6	1.33	0.54	6	1.46	0.37	6	1.88	0.47	6
**6**	1.25	0.25	3	1.17	0.26	6	1.21	0.33	6	1.33	0.20	6	1.50	0.42	6
**7**	0.75	0.25	3	0.88	0.61	6	1.25	0.84	6	0.71	0.43	6	0.88	0.72	6

SUPPLEMENTARY FIGURES

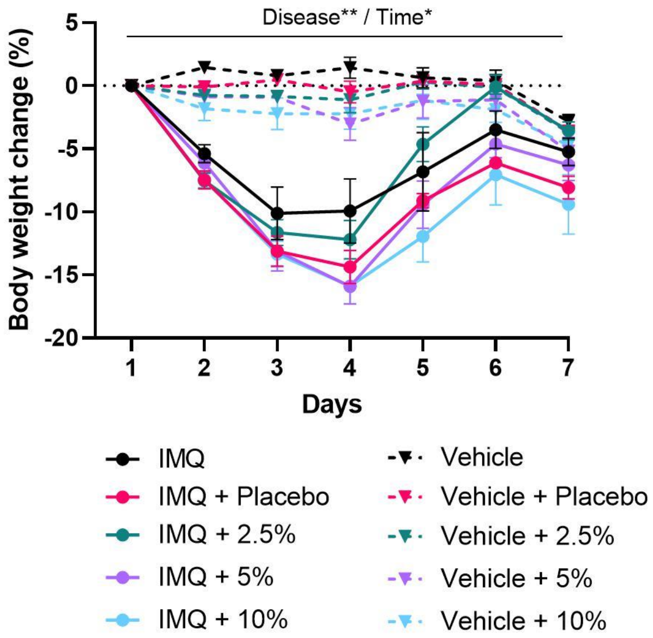

Supplementary Figure S1. Cumulative semiquantitative scores of clinical severity per day per treatment group. Body weight change (in % to starting weight) in mice in response to psoriasis induction by daily topical application of 62.5 mg Aldara® (IMQ) to the dorsal skin. Following IMQ, mice were treated with 2.5%, 5% or 10% diacerein or placebo or the skin was left untreated. Non-psoriatic control mice received vehicle cream. Data represent means ± SEM. n = 3-9 per group. Three independent experiments. *$p < 0.05$, **$p < 0.01$, repeated measures 2-way ANOVA main effects.

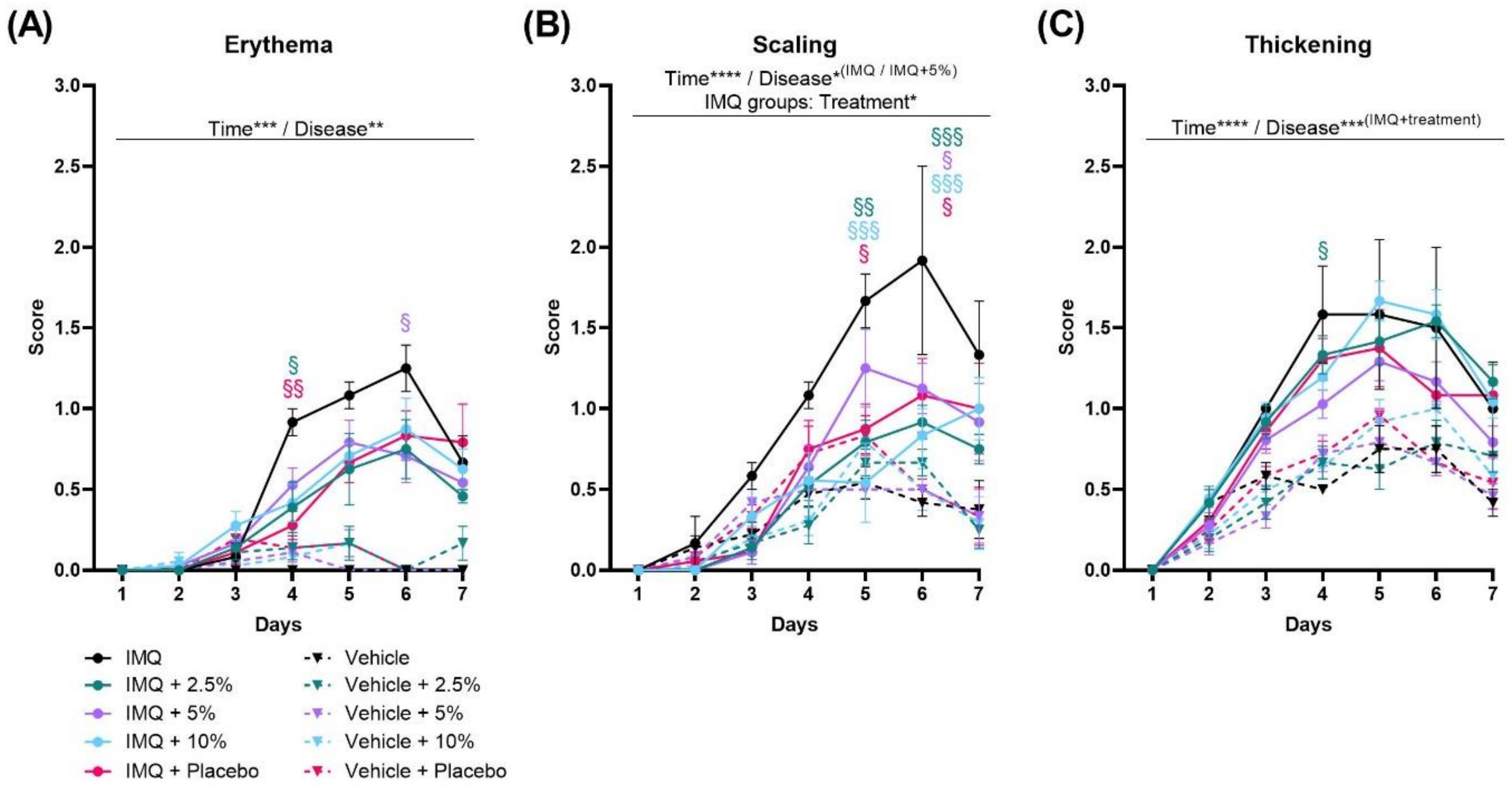

Supplementary Figure S2. Effect of diacerein on clinical symptoms of IMQ-induced psoriasis. Clinical severity was monitored daily by semiquantitative scoring of erythema (A), scaling (B) and thickening (C) of the dorsal skin. Psoriasis was induced by daily topical application of 62.5 mg Aldara® (IMQ) to the dorsal skin. Following IMQ, mice were treated with 2.5%, 5% or 10% diacerein or placebo or the skin was left untreated. Non-psoriatic control mice received vehicle instead of IMQ. Data represent means ± SEM. n = 3-9 per group. Three independent experiments. *p < 0.05, **p < 0.01, ***p < 0.001, ****p < 0.0001 repeated measures 2-way ANOVA main effects. §p < 0.05, §§p < 0.01, §§§p < 0.001 vs. untreated IMQ, Tukey's multiple comparison test.

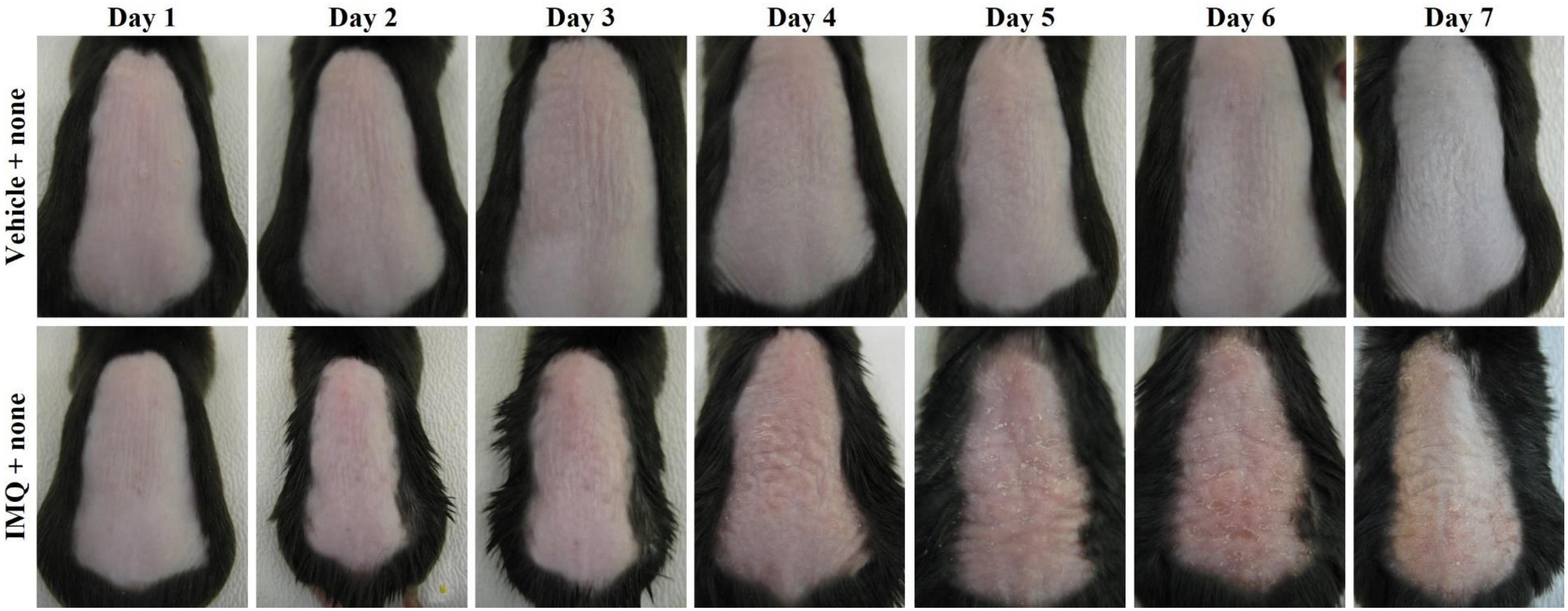

Supplementary Figure S3. Representative photographs of the daily documentation of the clinical severity of IMQ-induced psoriasis. Per treatment day, the treated dorsal skin is shown. The upper row shows mice treated with vehicle only. The lower row shows mice treated with 62 mg IMQ only.

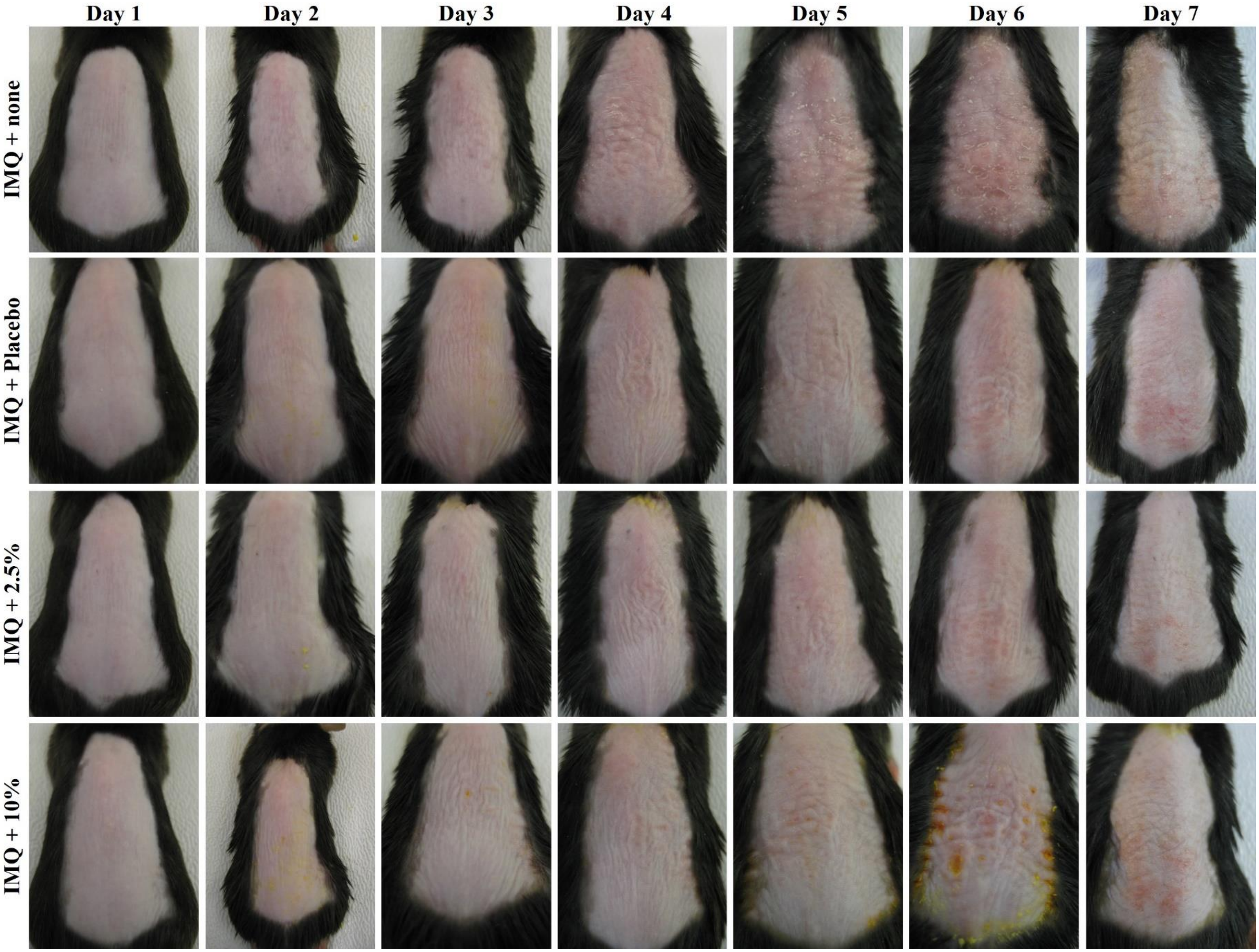

Supplementary Figure S4.

Supplementary Figure S4. Representative photographs of the daily documentation of the clinical severity of IMQ-induced psoriasis. Per treatment day, the treated dorsal skin is shown. The first row shows mice treated with 62 mg IMQ only. The second row shows mice treated with 62 mg IMQ, followed by Placebo. The third row shows mice treated with 62 mg IMQ, followed by 2.5% diacerein. The forth row shows mice treated with 62 mg IMQ, followed by 10% diacerein. On the dorsal skin of some mice, traces of the yellow-colored cream are visible.

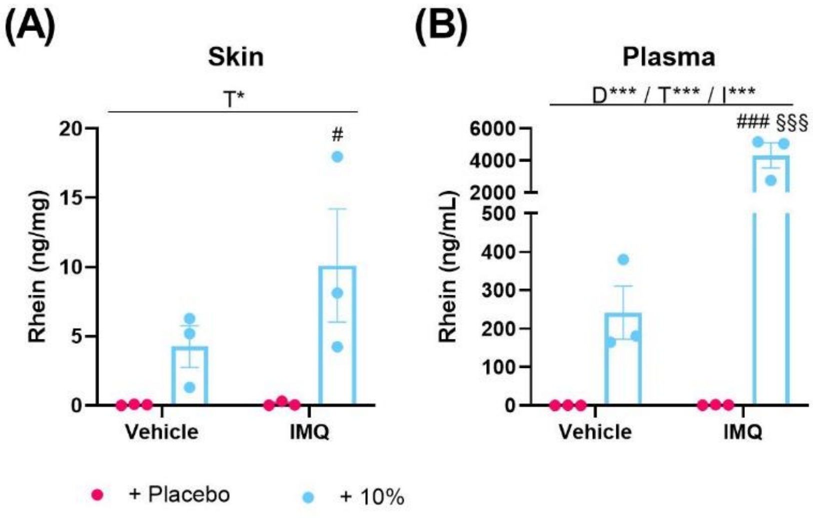

Supplementary Figure S5. Rhein levels in skin and plasma. Rhein, the active metabolite of diacerein, was quantified in an 8-mm biopsy of the treated area of the dorsal skin (A) and in plasma (B) on day 4. Psoriasis was induced by daily topical application of 62.5 mg Aldara® (IMQ) to the dorsal skin. Following IMQ, mice were treated with 10% diacerein or placebo. Non-psoriatic control mice received vehicle instead of IMQ. Data represent means ± SEM. n = 3 per group. *$p < 0.05$, ***$p < 0.001$, 2-way ANOVA main effects (D=disease, T=treatment, I=interaction). Tukey's multiple comparisons test: §§§$p < 0.001$ vs. respective vehicle group; #$p < 0.05$, ###$p < 0.001$ vs. IMQ + placebo.

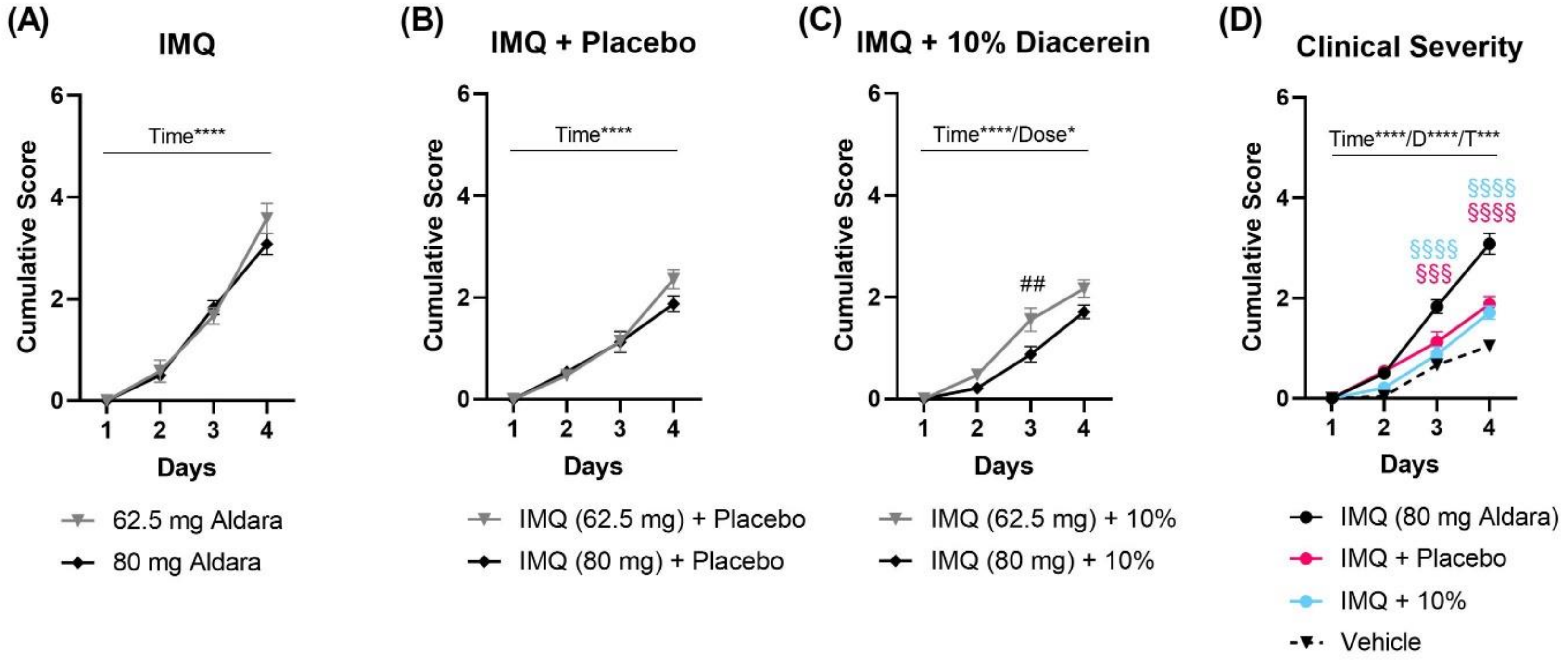

Supplementary Figure S6. Comparison of diacerein's effect on clinical symptoms following psoriasis induction with 62.5 or 80 mg Aldara®. Clinical severity is represented by the cumulative score calculated from daily semiquantitative score points for erythema, scaling and thickening of the dorsal skin. Comparison of the course of the clinical severity over 4 days after psoriasis induction with 62.5 mg or 80 mg Aldara® (A-C). After topical application of 62.5 mg (A-C) or 80 mg (A-D) Aldara® (IMQ), the dorsal skin was left untreated (A, D) or was treated with placebo (B, D) or 10% diacerein (C, D). Non-psoriatic control mice received vehicle only (D). Data represent means ± SEM. n = 3-9 (A-C), n = 6 (D). ***$p < 0.001$, ****$p < 0.0001$, 2-way ANOVA main effects (D=disease, T=treatment). Sidak's multiple comparisons test: ##$p < 0.01$; Tukey's multiple comparisons test: §§§$p < 0.001$, §§§§$p < 0.0001$ vs. IMQ.

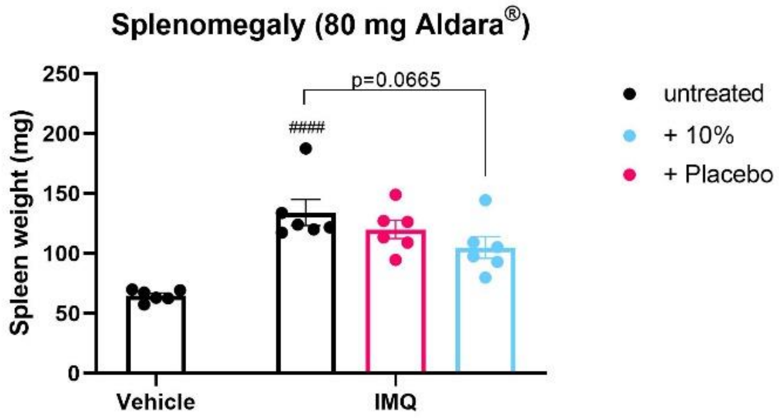

Supplementary Figure S7. Effect of diacerein on splenomegaly following psoriasis induction with 80 mg Aldara®. Spleen weight in mice on day 4 after receiving treatment for 3 consecutive days. Psoriasis was induced by daily topical application of 80.0 mg Aldara® (IMQ) to the dorsal skin. Following IMQ, mice were treated with 100 mg of 10% diacerein or placebo or the skin was left untreated. Non-psoriatic control mice received vehicle only. Data represent means ± SEM. n = 6 per group. ####$p < 0.0001$ unpaired t-test, vehicle vs. untreated IMQ.

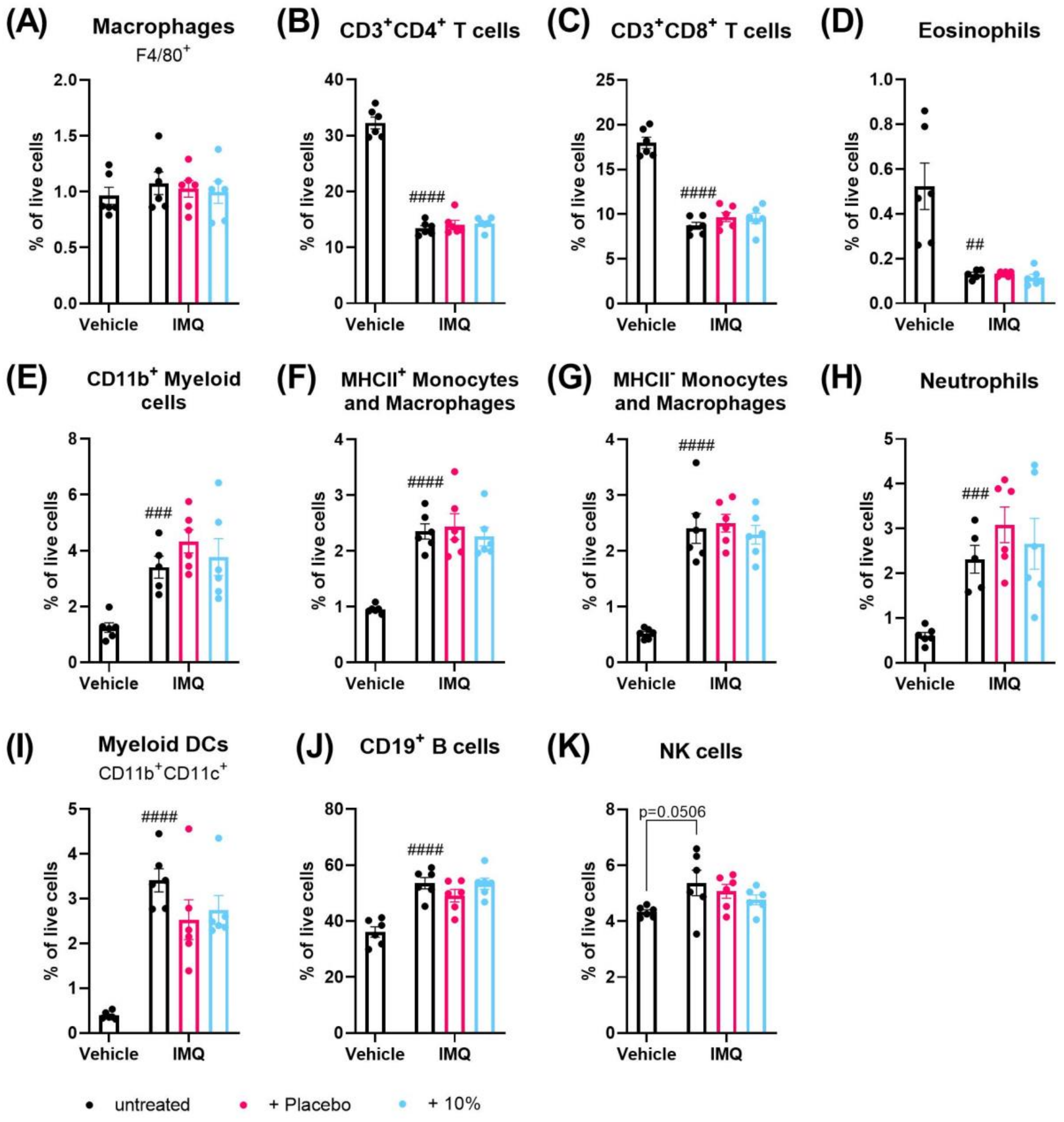

Supplementary Figure S8.

Supplementary Figure S8. Splenic immune cell populations which are unaffected by diacerein treatment. Flow cytometric analysis of F4/80^{+} macrophages (A), CD4^{+} (B) and CD8^{+} T cells (C), eosinophils (D), CD11b^{+} myeloid cells (E), MHCII^{+} (F) and MHCII^{-} (G) monocytes/macrophages, neutrophils (H), CD11b^{+}CD11c^{+} myeloid dendritic cells (DCs) (I), CD19^{+} B cells (J) and NK cells (K) in spleen single cell suspensions of mice on day 4 after receiving treatment for 3 consecutive days. Psoriasis was induced by daily topical application of 80.0 mg Aldara® (IMQ) to the dorsal skin. Following IMQ, mice were treated with 100 mg of 10% diacerein or placebo or the skin was left untreated. Non-psoriatic control mice received vehicle only. Data represent means ± SEM. n = 5-6 per group. ###p < 0.001, ####p < 0.0001 unpaired t-test, vehicle vs. untreated IMQ.

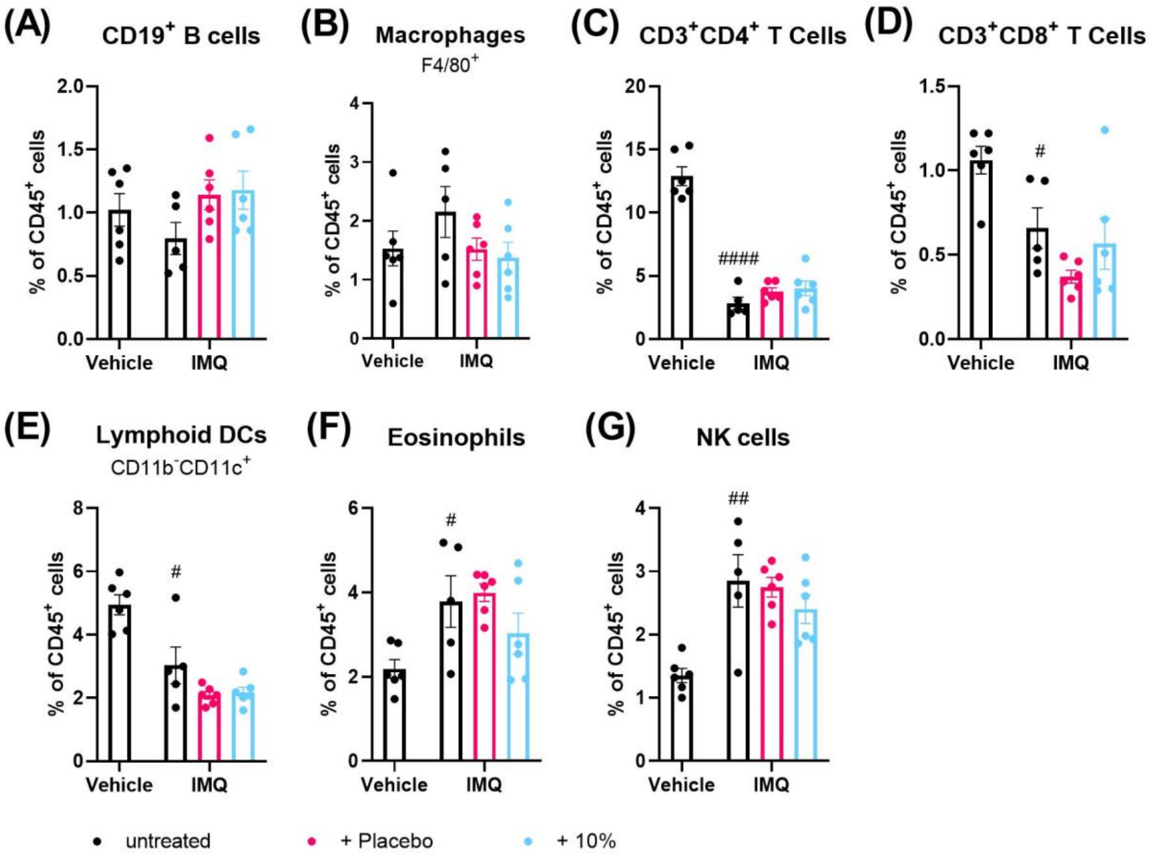

Supplementary Figure S9. Skin immune cell populations which are unaffected by diacerein treatment. Flow cytometric analysis of $CD19^+$ B cells (A), $F4/80^+$ macrophages (B), $CD4^+$ (C) and $CD8^+$ T cells (D), $CD11b^-CD11c^+$ lymphoid dendritic cells (DCs) (E), eosinophils (F) and NK cells (G) in skin single cell suspensions of mice on day 4 after receiving treatment for 3 consecutive days. Psoriasis was induced by daily topical application of 80.0 mg Aldara® (IMQ) to the dorsal skin. Following IMQ, mice were treated with 100 mg of 10% diacerein or placebo or the skin was left untreated. Non-psoriatic control mice received vehicle only. Data represent means ± SEM. n = 5-6 per group. #$p < 0.05$, ##$p < 0.01$, unpaired t-test, vehicle vs. untreated IMQ.

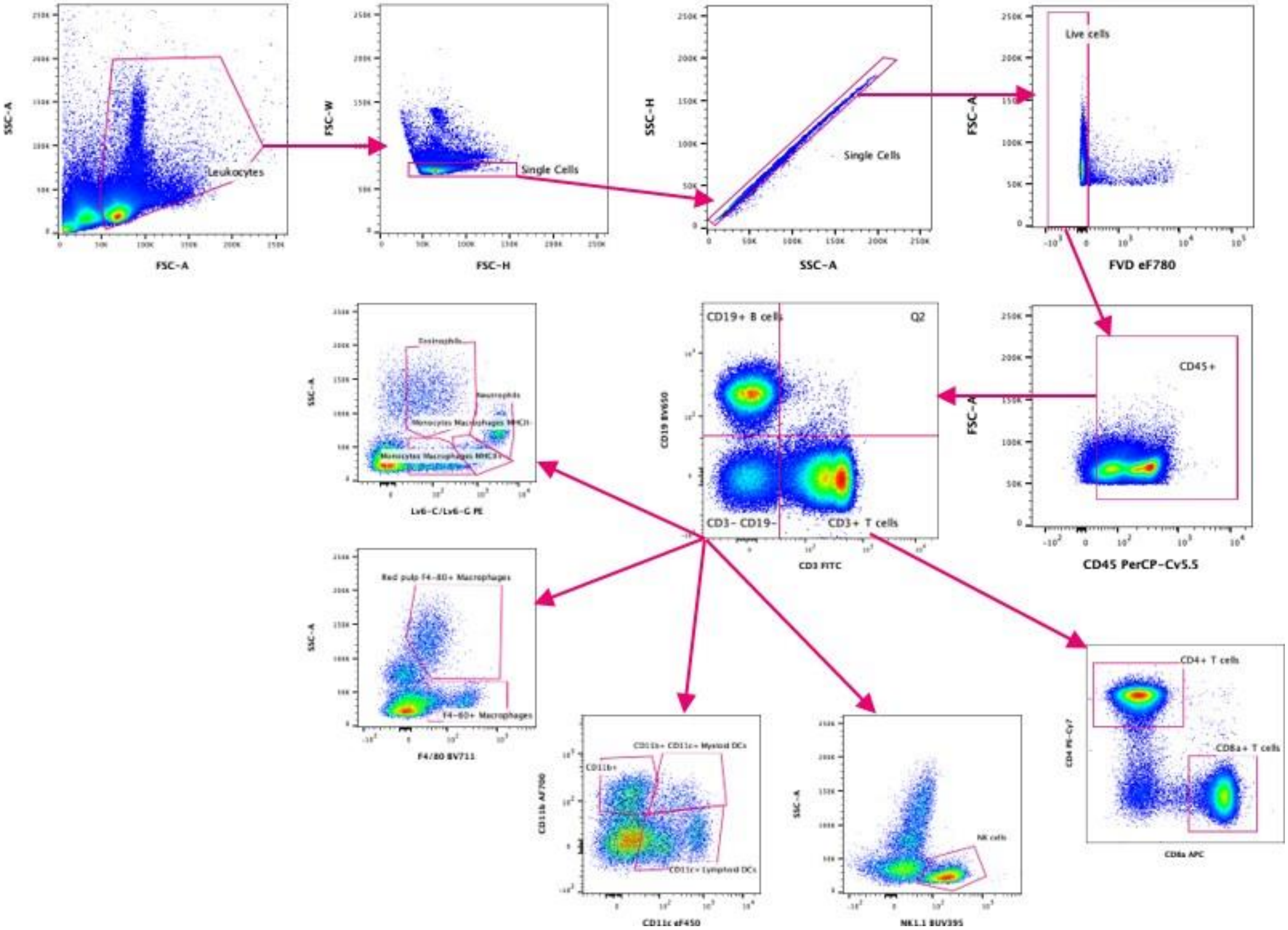

Supplementary Figure S10.

Supplementary Figure S10. Gating strategy for flow cytometric analysis of diverse immune cell populations and subsets in the spleen. Representative flow plots of a spleen sample are shown. Leukocytes were gated based on forward (FSC) and side scatter (SSC) profiles followed by exclusion of doublets and selection for live cells using a fixable viability dye (FVD). Further, cells exhibiting CD45 positivity were selected. From this population, CD3 and CD19 expression was used to define $CD19^{+}CD3^{-}$ B cells and $CD19^{-}CD3^{+}$ T cells and subsequently $CD4^{+}$ and $CD8^{+}$ T cells. The $CD19^{-}CD3^{-}$ population was further gated to identify (i) $F4/80^{+}$ macrophages and red pulp macrophages (RPMs) based on F4/80 positivity and SSC profiles (ii) eosinophils, neutrophils, $MHCII^{+}$ and $MHCII^{-}$ monocytes and macrophages based on Ly-6C/Ly-6G positivity and SSC profiles (iii) $CD11b^{+}CD11c^{-}$ myeloid cells, $CD11b^{+}CD11c^{+}$ myeloid dendritic cells (DCs) and $CD11b^{-}CD11c^{+}$ lymphoid DCs based on CD11b and CD11c positivity and (iv) NK cells based on NK1.1 positivity. Setting of the gates was performed as described previously [1-5] Used antibodies and conjugated fluorophores are summarized in Table 1.

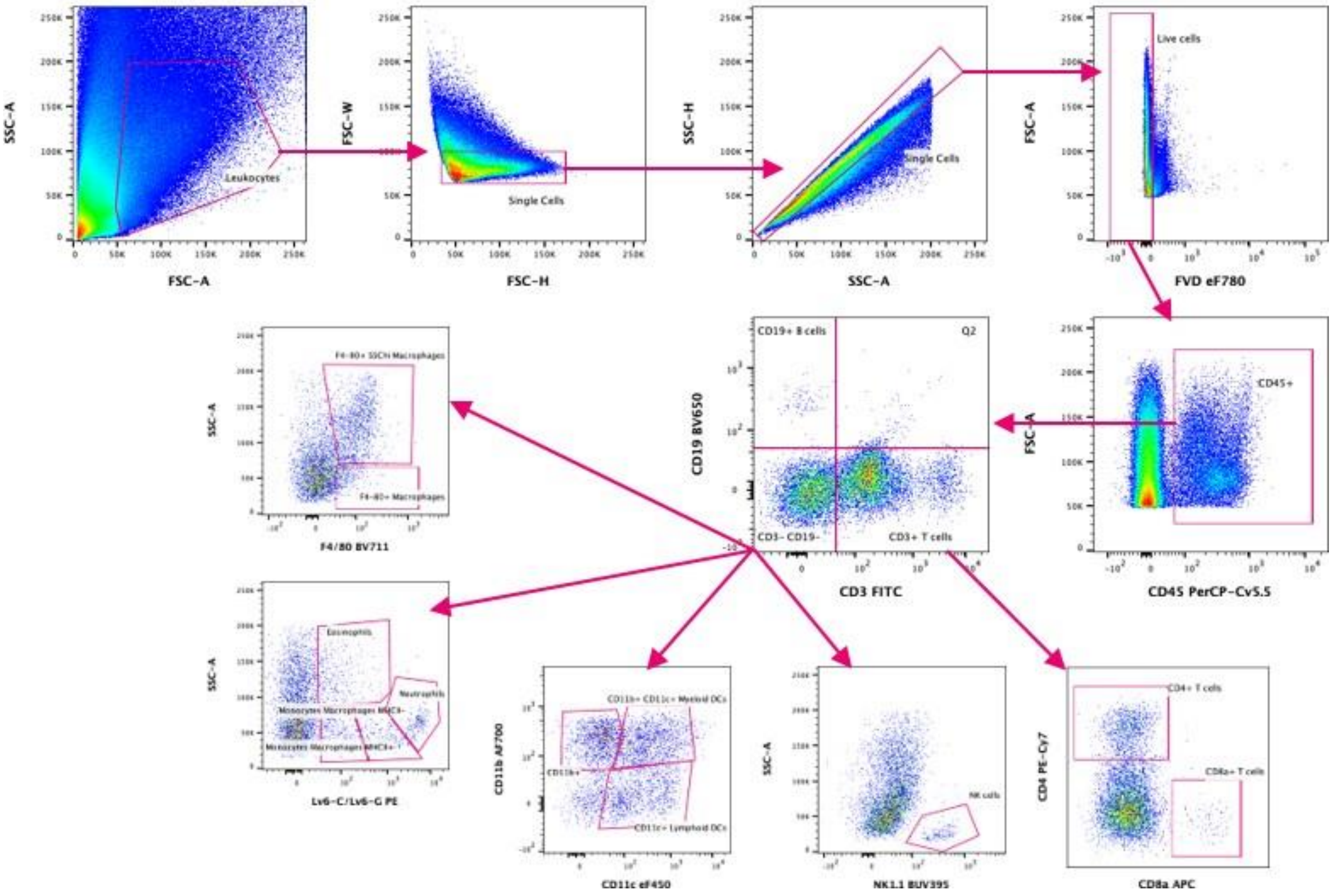

Supplementary Figure S11.

Supplementary Figure S11. Gating strategy for flow cytometric analysis of diverse immune cell populations and subsets in the dorsal skin. Representative flow plots of a skin sample are shown. Leukocytes were gated based on forward (FSC) and side scatter (SSC) profiles followed by exclusion of doublets and selection for live cells using a fixable viability dye (FVD). Further, cells exhibiting CD45 positivity were selected. From this population, CD3 and CD19 expression was used to define $CD19^{+}CD3^{-}$ B cells and $CD19^{-}CD3^{+}$ T cells and subsequently $CD4^{+}$ and $CD8^{+}$ T cells. The $CD19^{-}CD3^{-}$ population was further gated to identify (i) $F4/80^{+}$ macrophages and red pulp macrophages (RPMs) based on F4/80 positivity and SSC profiles (ii) eosinophils, neutrophils, $MHCII^{+}$ and $MHCII^{-}$ monocytes and macrophages based on Ly-6C/Ly-6G positivity and SSC profiles (iii) $CD11b^{+}CD11c^{-}$ myeloid cells, $CD11b^{+}CD11c^{+}$ myeloid dendritic cells (DCs) and $CD11b^{-}CD11c^{+}$ lymphoid DCs based on CD11b and CD11c positivity and (iv) NK cells based on NK1.1 positivity. Setting of the gates was performed as described previously [1-5] Used antibodies and conjugated fluorophores are summarized in Table 1.

SUPPLEMENTARY REFERENCES

1. Palamara, F., et al., *Identification and characterization of pDC-like cells in normal mouse skin and melanomas treated with imiquimod.* J Immunol, 2004. **173**(5): p. 3051-3061.
2. Lou, F., Y. Sun, and H. Wang, *Protocol for Flow Cytometric Detection of Immune Cell Infiltration in the Epidermis and Dermis of a Psoriasis Mouse Model.* STAR Protoc, 2020. **1**(3): p. 100115.
3. Yu, Y.R., et al., *A Protocol for the Comprehensive Flow Cytometric Analysis of Immune Cells in Normal and Inflamed Murine Non-Lymphoid Tissues.* PLoS One, 2016. **11**(3): p. e0150606.
4. Rose, S., A. Misharin, and H. Perlman, *A novel Ly6C/Ly6G-based strategy to analyze the mouse splenic myeloid compartment.* Cytometry A, 2012. **81**(4): p. 343-350.
5. Liu, Z., et al., *Analysis of Myeloid Cells in Mouse Tissues with Flow Cytometry.* STAR Protoc, 2020. **1**(1): p. 100029.
